# Conceptual design of the energy-switchable storage ring as a high-brilliance light source over a wide wavelength range

**DOI:** 10.1107/S1600577525005363

**Published:** 2025-07-21

**Authors:** Tomoko Sato, Nobumasa Funamori, Kenta Amemiya, Noriko Usami, Takuji Ohigashi, Ryoma Kataoka, Nobutaka Shimizu, Hirokazu Tanaka, Hironori Nakao, Yusuke Yamada, Daisuke Wakabayashi, Takashi Obina, Masahiro Adachi, Yukinori Kobayashi, Yoshito Shimosaki, Yasunori Tanimoto, Kimichika Tsuchiya, Kentaro Harada, Naoto Yamamoto, Noriyuki Igarashi

**Affiliations:** a Institute of Materials Structure Science High Energy Accelerator Research Organization (KEK) 1-1 Oho Tsukuba Ibaraki305-0801 Japan; b Accelerator Laboratory High Energy Accelerator Research Organization (KEK) 1-1 Oho Tsukuba Ibaraki305-0801 Japan; RIKEN SPring-8 Center, Japan

**Keywords:** light source, energy switchable, efficient power consumption, wide-wavelength range application

## Abstract

The energy-switchable storage ring (ESSR) is proposed as a light source that achieves high-brilliance synchrotron radiation across a wide wavelength range, from vacuum ultraviolet to hard X-rays, and efficient power consumption. The ESSR facilitates the effective operation of large-scale systems required for large experimental apparatus and stringent safety management. It also significantly improves research quality by enabling a more precise understanding of sample states, particularly subtle differences in experimental conditions that are difficult to reproduce fully.

## Abstract 2

The concept of the energy-switchable storage ring, designed for wide-wavelength range applications and efficient power consumption, is presented.

## 1. Energy of synchrotron light sources

Synchrotron radiation, discovered in the mid-20th century, has proven invaluable for a broad spectrum of materials and life sciences research. As a result, electron storage rings have been constructed globally and are utilized as essential research infrastructure. These storage rings can be broadly classified into three categories: small rings for circulating low-energy electrons (< ∼1.5 GeV), medium rings for mid-range energy (∼3 GeV) and large rings for high-energy electrons (> ∼6 GeV). Each type excels at different wavelength ranges—vacuum ultraviolet, soft X-rays and hard X-rays, respectively—and accommodates a wide wavelength range through various insertion devices designed for their respective specialized wavelengths.

Typically, storage rings are designed and operated at a fixed electron energy. However, by changing the magnetic field of the electromagnets, it is possible to switch the electron energy within the same ring. For example, at Photon Factory (PF), with which the authors are affiliated, the PF-AR storage ring can be operated at both 5.0 GeV and 6.5 GeV. Operation at 5.0 GeV reduces the power consumption, whereas operation at 6.5 GeV enhances the short-wavelength performance. Other examples include Elettra, which operates at 2.0/2.4 GeV, and NewSUBARU, which operates at 1.0/1.5 GeV.

For further advancements in synchrotron radiation science, it is crucial to meet existing demands and foster the development of new research seeds. Nurturing these seeds is closely tied to the cultivation of future talent. To support the development of diverse research seeds, versatility in the storage ring design is essential, a concept shared by the hybrid ring (Harada *et al.*, 2022 ▸), which we previously proposed. Simultaneously, reducing power consumption has become a global challenge. This paper proposes a high-brilliance storage ring with unprecedented energy-switching capabilities in a short time, allowing operation over a wide range of wavelengths, from vacuum ultraviolet to hard X-rays. The discussion covers the technical challenges, their potential solutions, and new scientific possibilities that such a design could unlock.

## 2. Concept of the energy-switchable storage ring

The energy-switchable storage ring (ESSR) is proposed as a light source that achieves high-brilliance synchrotron radiation across a wide wavelength range, from vacuum ultraviolet to hard X-rays, and reduced power consumption. Globally, there is a trend toward reducing the emittance (increasing brilliance) of storage rings. The ESSR builds on this trend by further expanding the usable wavelength range, aiming to serve as a versatile light source that meets the diverse needs of materials and life sciences. With a medium-to-large ring circumference, the ESSR design includes numerous straight sections and a variety of insertion devices, enabling the use of high-brilliance light across a wide wavelength range by switching the electron energies. Furthermore, the ability to switch energies quickly enables diverse measurements of the same sample, contributing to the advancement of new scientific explorations. While worldwide efforts are focused on reducing power consumption by adapting permanent magnets instead of electromagnets (Shepherd, 2020 ▸; Raimondi *et al.*, 2023 ▸; Tanaka *et al.*, 2024 ▸), the ESSR retains the flexibility to change the magnetic field through electromagnets, thus allowing it to cover high-demand wavelength ranges during low-energy operation while setting the duration of high-energy operation to the bare minimum. Consequently, the ESSR provides high-brilliance light across a wide wavelength range with reduced power consumption. Although the overall construction cost for the ESSR with a large circumference is higher than that for rings with a small to medium circumference, the cost per beamline is comparable, with significant improvements in performance (brilliance and wavelength range).

### 2.1. Parameters and spectra

**Figure 1:**
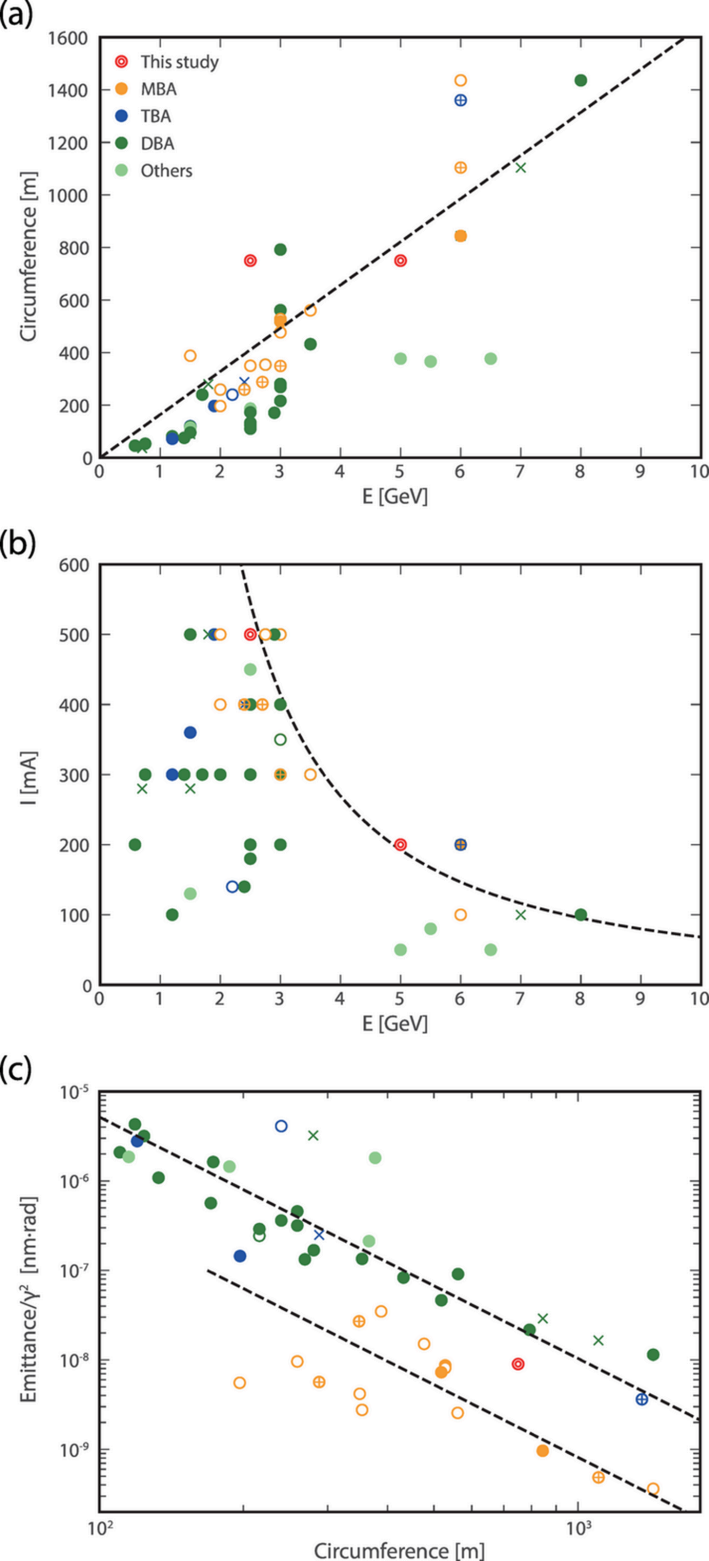
(*a*) Relation between electron energy and circumference, (*b*) relation between electron energy and storage current, and (*c*) the natural emittance of synchrotron radiation facilities in the world. The dashed lines serve as a guide for the eyes, but in case (*b*) it follows *I* ∝ *E*^−1.5^ and in case (*c*) they satisfy ∝ γ^2^/*C*^3^. Solid circles, × marks, crosses with circle, open circles and double circles indicate under operation, halted operation, construction, planning and this study, respectively.

The tentative parameters for the ESSR are listed in Table 1 ▸. To accommodate a wide wavelength range spanning from vacuum ultraviolet to hard X-rays, the electron energy is set to 2.5/5.0 GeV. For synchrotron facilities worldwide, the relationship between electron energy and ring circumference is approximately proportional, mainly because of the magnetic field strength limitations of the bending magnets [Fig. 1 ▸(*a*)]. The circumference of the ESSR is therefore set to 750 m to support 5.0 GeV operation. Additionally, due to constraints related to the radiation power and beam instabilities, the maximum storage current is approximately inversely proportional to the 1.5th power of the electron energy [Fig. 1 ▸(*b*)]. Following this trend, the storage current is set to 500 mA at 2.5 GeV and 200 mA at 5.0 GeV. The emittance is appropriately low for the given circumference [Fig. 1 ▸(*c*)]. Operating the ring at 2.5 GeV with a 750 m circumference, suited for 5.0 GeV, is advantageous in terms of emittance compared with a ring optimized for 2.5 GeV.

**Figure 2:**
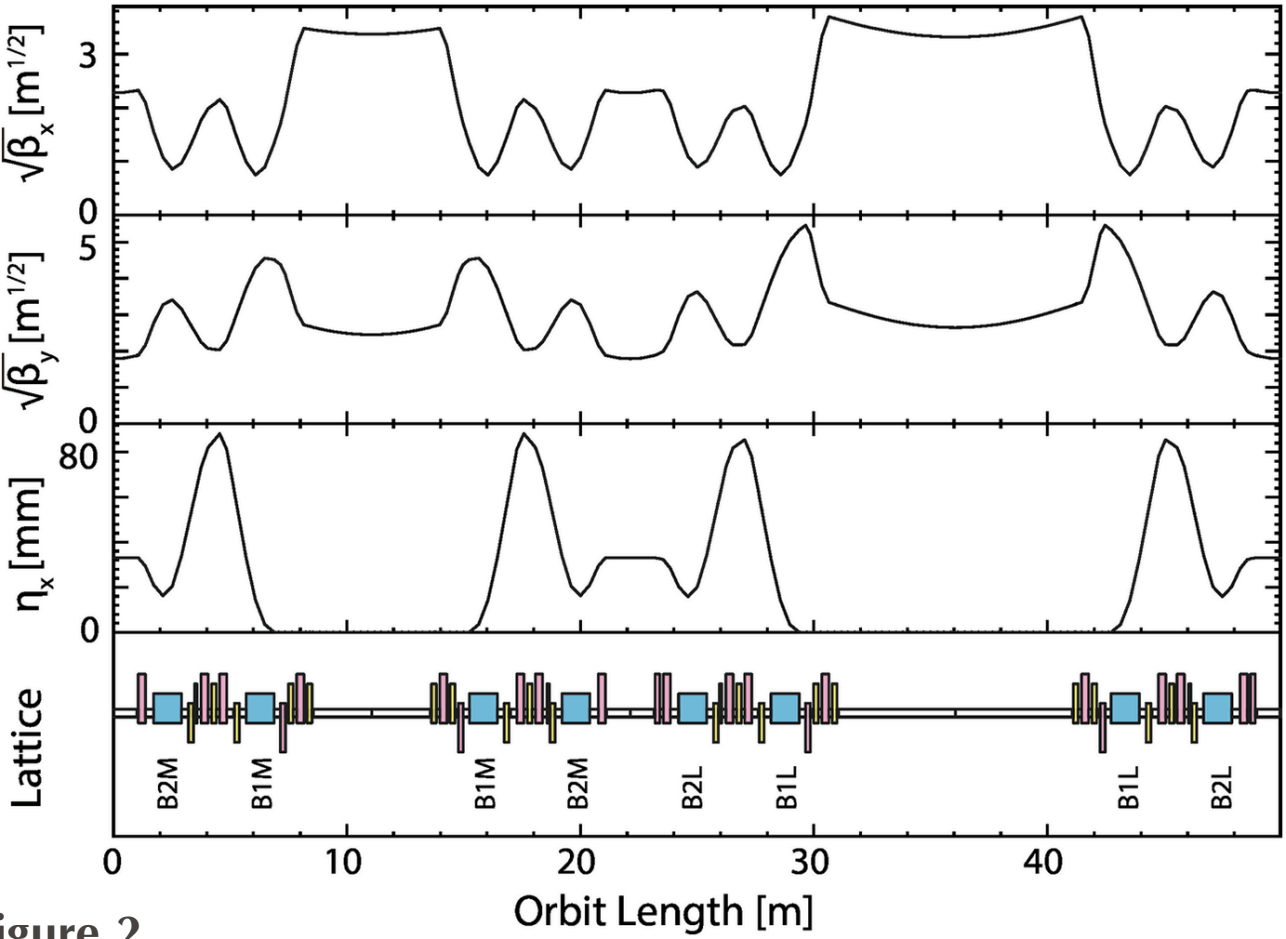
Optics and lattice of the ESSR.

The lattice design is based on the double-double-bend achromat (DDBA) employed by NanoTerasu, which recently began operations in Sendai, Japan (Nishimori *et al.*, 2019 ▸). Each cell includes one 10 m and one 5 m straight section, as well as two 2 m straight sections (Fig. 2 ▸). The 10 m long straight section is designed to accommodate multiple insertion devices, enabling the delivery of a wide wavelength range of synchrotron radiation to a single beamline and facilitating previously proposed multi-beam applications (Harada *et al.*, 2022 ▸). Although placing a 10 m straight section presents challenges in beam optics, sufficient dynamic aperture has been achieved through careful optimization of sextupole magnets.

**Figure 3:**
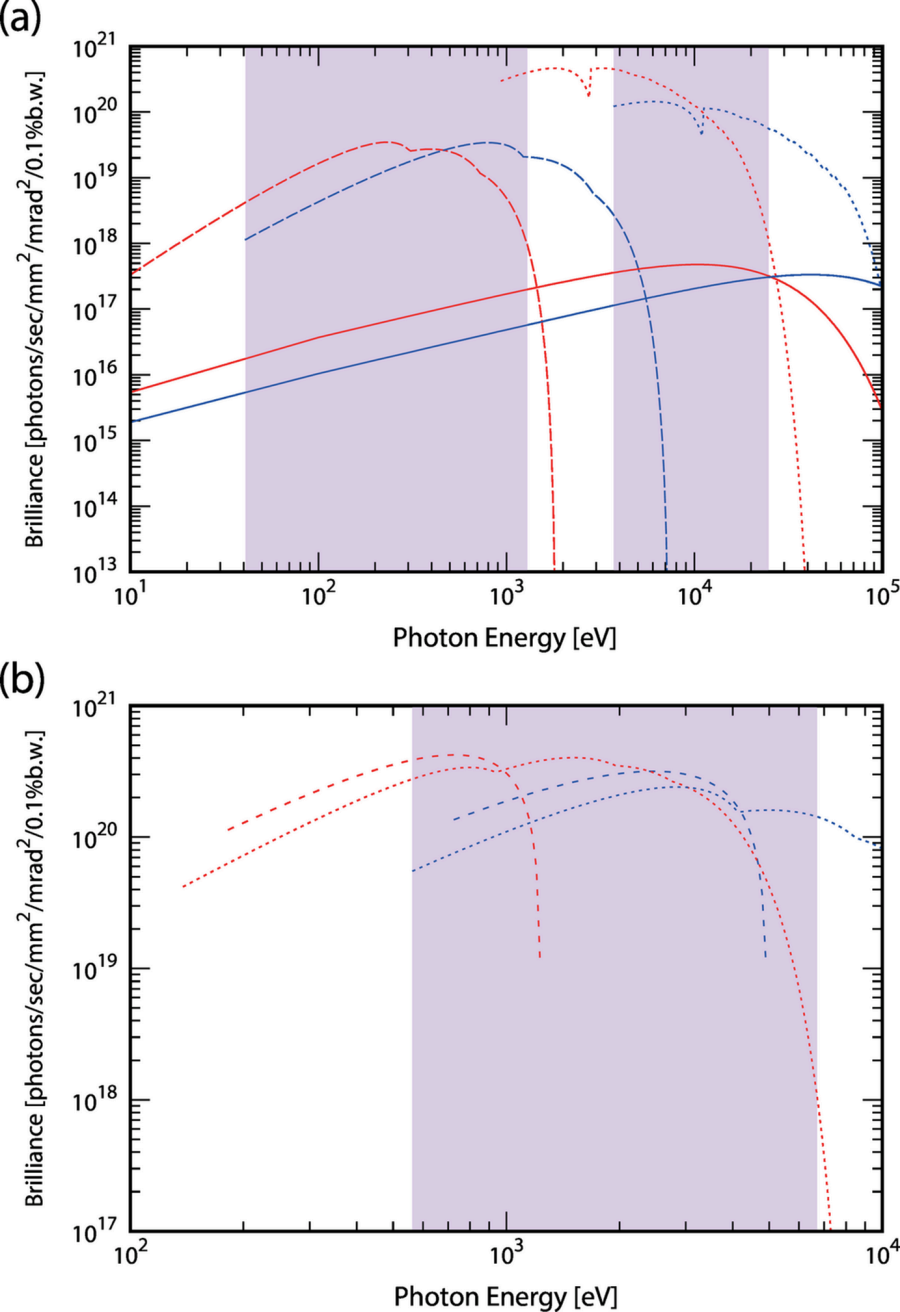
Brilliance of the typical insertion devices operated at 2.5 GeV (red lines) and 5.0 GeV (blue lines). The areas highlighted in purple indicate the wavelength range available for both 2.5 GeV and 5.0 GeV. (*a*) Spectra from the six-row type variable polarization type undulator (1st–5th harmonics), in-vacuum undulator (1 m, 1st–15th) and superconducting multipole wiggler, in broken, dotted and solid lines, respectively, and (*b*) those from the APPLE-II type variable polarization undulator, broken and dotted lines for circular (1st) and linear (1st–5th) polarization modes, respectively.

The parameters and brilliance spectra of typical insertion devices are provided in Table 2 ▸ and Fig. 3 ▸. Using the same insertion device, switching from 2.5 GeV to 5.0 GeV results in a fourfold increase in the photon energy. As shown in Fig. 3 ▸(*a*), installing both an in-vacuum undulator and a six-row type elliptical undulator in a 10 m straight section enables the delivery of high-brilliance light with photon energies ranging from 10 eV to 100 keV on a single beamline. Additionally, as shown in Fig. 3 ▸(*b*), a fundamental harmonic of an Apple-II-type elliptical undulator, commonly used for generating circularly polarized soft X-rays, can cover a wide energy range from 200 eV to 4 keV.

### 2.2. Power consumption

A simplified model of the power consumption of a synchrotron radiation facility *P*_facility_ is considered as follows. This model assumes that the total power consumption is the sum of the power consumption for the electromagnets *P*_magnet_, radio-frequency (RF) cavities *P*_RF_, other power consumption during operation *P*_others_run_, and the power consumption common to both the operating and shutdown periods *P*_others_base_, 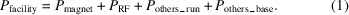
The magnetic field *B* [T] of the bending magnet is given by the electron energy *E* [GeV] and the bending radius ρ [m] (Wiedemann, 1993 ▸),
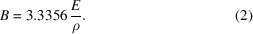
Assuming that the current supplied to the bending magnets is proportional to *B*, the number of bending magnets *n* is proportional to the circumference *C* [m], *C* is proportional to ρ, and further assuming that the power consumption of other types of magnets, such as quadrupoles and sextupoles, is proportional to the power consumption of the bending magnets, the expression for the magnet power consumption can be written as
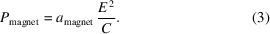
The radiation loss *U* can be divided into the loss from the bending magnets *U*_B_ and the loss from insertion devices *U*_I_. *U*_B_ is proportional to the fourth power of *E* and inversely proportional to ρ (Wiedemann, 1993 ▸). The radiation loss from insertion devices is proportional to the square of *E* (Wiedemann, 1995 ▸). While *U*_I_ depends on the type and number of installed insertion devices, considering only the case in which the number of insertion devices is proportional to the circumference, the power consumption of the RF cavities can be expressed using the storage current *I* [mA] as follows,
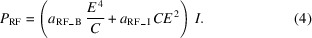
During accelerator operation, it is necessary to remove the heat generated, and it is assumed that the required power consumption is proportional to *P*_magnet_ + *P*_RF_. Additionally, even during shutdown, power is required for building air conditioning and other essential services. This common power consumption during both operation and shutdown is considered to be proportional to the size of the facility, which in turn scales with *C*, assuming that the radial width of the ring remains constant regardless of the circumference. Thus, the other power consumption is given by
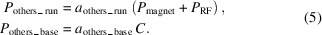
The difference in the power consumption of the beamline equipment during operation and shutdown is assumed to be negligible. The power consumption of an injector varies significantly depending on the injection scheme employed. For example, in the case of a booster, if it is designed to minimize power consumption except during injection, its average power consumption can be considerably lower than that of the storage ring. Therefore, the injector is excluded from the scope of this discussion. Based on information from PF and PF-AR facilities operated by the authors, along with data from SLS, ESRF and SPring-8 (Table S1 of the supporting information), the coefficients *a*_magnet_, *a*_RF_B_, *a*_RF_I_, *a*_others_run_ and *a*_others_base_ were estimated to be 50, 1 × 10^−2^, 1 × 10^−7^, 0.4 and 3 × 10^−3^, respectively, with the power consumption expressed in MW. Here, the units of these coefficients are omitted because they do not have physical meaning. The ratio of *a*_RF_B_ to *a*_RF_I_ was estimated based on the design values for *U*_B_ and *U*_I_ published by various facilities.

**Figure 4:**
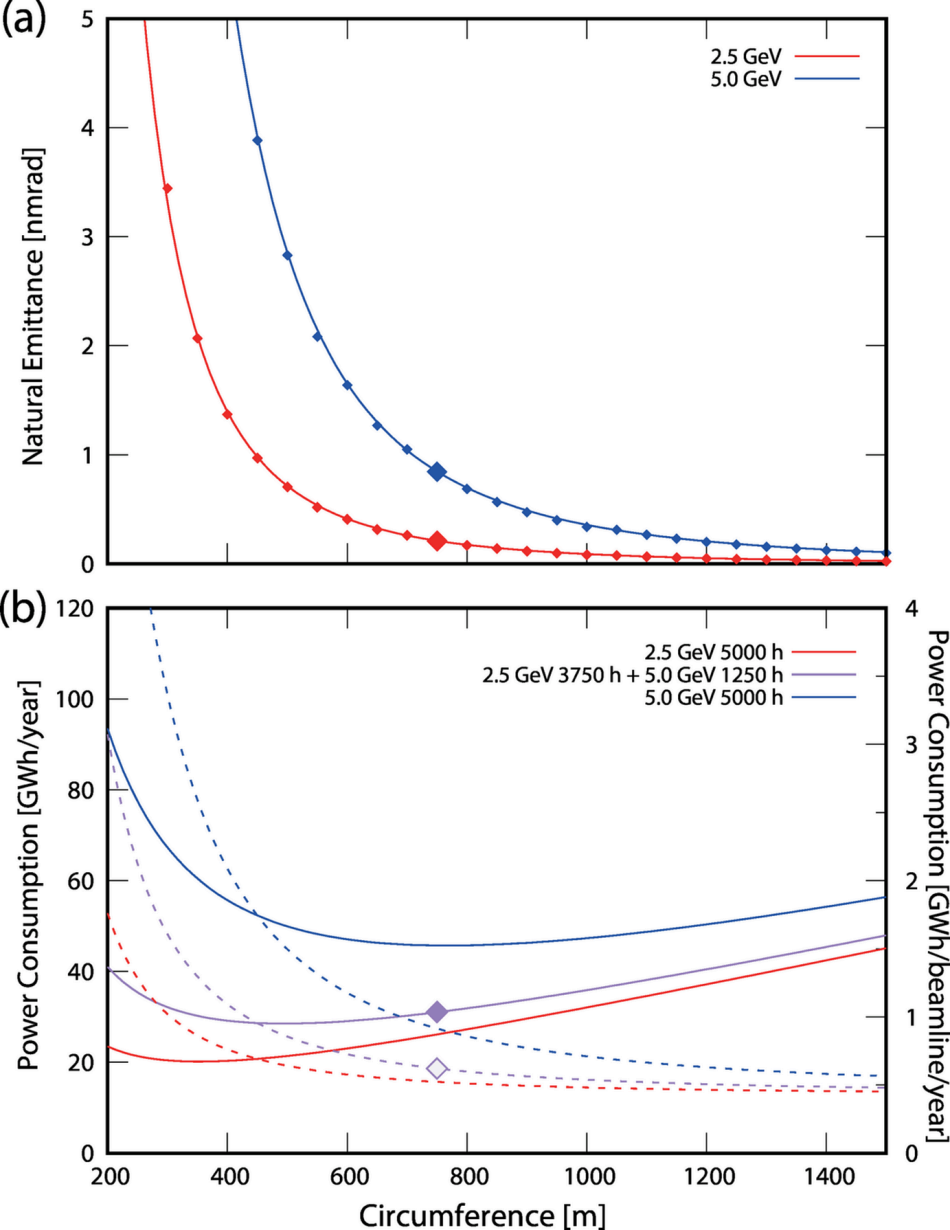
(*a*) Dependence of the horizontal emittance on the circumference. The points represent the values when the number of cells is varied, and the lines represents the values inversely proportional to the cube of the circumference, based on the value at 750 m. (*b*) Estimation of power consumption for the entire facility and per individual beamline in solid and dotted lines, respectively. For the latter, the estimation assumed that one beamline can be installed for every 15 m of circumference.

Although there is significant uncertainty in the values of these coefficients owing to the lack of public data and the variation in equipment and operation across facilities, this model is sufficient to understand the approximate dependence of power consumption on energy and circumference. According to this model, the power consumption of a storage ring with a circumference of 750 m is estimated to be 3.5 MW during 2.5 GeV/500 mA operation, 7.4 MW during 5.0 GeV/200 mA operation, and 2.3 MW during shutdown. Assuming the use of permanent magnets, an estimation of the power consumption during 5.0 GeV operation excluding that of electromagnets results in 5.1 MW, which is higher than that during 2.5 GeV operation with electromagnets. Therefore, minimizing the operation time at 5.0 GeV is an effective strategy for reducing power consumption. Assuming 5000 h of operation per year, the total energy consumption would be 26 GW h if operated entirely at 2.5 GeV and 46 GW h at 5.0 GeV. For example, if 2.5 GeV operation is performed for 3750 h and 5.0 GeV operation is performed for 1250 h, the total energy consumption would be 31 GW h. In both 2.5 GeV and 5.0 GeV operations, high brilliance light can be used within the wavelength range indicated in purple in Fig. 3 ▸. This range includes the high-demand region of approximately 100 eV to 10 keV, and utilizing this range through the 2.5 GeV operation helps reduce the power consumption. The adoption of undulators covering a wider energy range (Bertwistle *et al.*, 2016 ▸; Bahrdt & Gluskin, 2018 ▸) may also be effective in further reducing high-energy operation. The number of beamlines that can be installed is approximately proportional to the circumference. Fig. 4 ▸(*a*) shows the dependence of the natural emittance on the circumference, and Fig. 4 ▸(*b*) illustrates the dependence of the total facility power consumption and power consumption per beamline on the circumference.

## 3. Light source and beamline of the ESSR

In this section, we outline the design principles of the ESSR. The ESSR must primarily be designed for high-energy operation to satisfy the demand of magnetic fields, accelerating voltage, cooling power, *etc*. The switching of the electron energy significantly affects the stability of the electron beam (and the associated synchrotron radiation), necessitating specific countermeasures. Furthermore, beamlines that receive synchrotron radiation over a wide wavelength range require appropriate arrangements. The following sections detail the key concerns associated with energy-switching operations and the strategies for addressing them.

### 3.1. Storage ring

The tentative parameters and lattice design of the 2.5/5.0 GeV ESSR are presented in Table 1 ▸ and Fig. 2 ▸, respectively. Switching between 2.5 GeV and 5.0 GeV operation is achieved by changing the operating current of the electromagnets. At 5.0 GeV, the current approaches the maximum output of the power supply, and the magnetic field is expected to enter the saturation region. In contrast, at 2.5 GeV, the impact of magnetic field saturation is minimal, and the current is less than half of that required for 5.0 GeV operation. Operating at a current lower than the maximum rating of the power supply poses no issues in terms of current stability; however, it has the drawback of reduced power efficiency. On the other hand, rapid energy switching requires the same set of power supplies. Therefore, installing multiple sets of power supplies should be considered depending on the application. To achieve a versatile light source accommodating a wide range of experiments, the minimum bore diameter of the electromagnets is set to 30 mm, the same as that of the previously proposed hybrid ring (Harada *et al.*, 2022 ▸). The magnetic field of the bending magnets is set relatively low at 0.7 T for 5.0 GeV. The magnetic field gradients are 52 T m^−1^ for the quadrupole magnets and 6400 T m^−2^ for the sextupole magnets at 5.0 GeV, both of which are feasible values.

Regarding the RF system, it is essential to ensure sufficient acceleration voltage and RF power for 5.0 GeV operation and to ensure sufficient beam stability during 2.5 GeV operation. To achieve the required accelerating voltage, sufficient total shunt impedance is introduced using multiple cavities. The cavities required for 5.0 GeV operation but not needed for 2.5 GeV operation will be put into standby mode by turning off their power supplies and detuning the resonant frequency. However, even in standby mode, the harmful parasitic modes (PMs) can contribute to coupled-bunch beam instabilities (CBI) (Akai, 1999 ▸). Therefore, cavities having a sufficiently low beam-coupling impedance of PMs should be employed. Considering maintainability, the normal-conducting accelerating cavities used for the SuperKEKB positron damping ring (DR) (SuperKEKB, 2020 ▸) become a candidate for the main RF cavities of the 2.5/5.0 GeV ESSR. The growth rates of the CBI caused by their total beam-coupling impedances in the longitudinal, horizontal and vertical directions at 2.5 GeV were estimated to be 183, 495 and 76 s^−1^, respectively, under the worst-case scenario, in which the resonant frequency of the PMs become a harmonic frequency of the beam revolution. For comparison, the growth rates for the EU cavities (500 MHz) (Weihreter, 2008 ▸), widely adopted as PM-damped cavities in single-energy storage rings, were estimated to be 798, 997 and 635 s^−1^, respectively. The SuperKEKB DR cavities exhibit remarkable performance against PMs. Although the estimated CBI growth rates still exceed the radiation damping rates, these instabilities are expected to be sufficiently suppressed by implementing countermeasures such as a bunch-by-bunch feedback system dedicated for both the transverse and longitudinal directions (Takai *et al.*, 2009 ▸; Tobiyama *et al.*, 2019 ▸). Alternatively, superconducting cavities are potential candidates. Assuming the KEKB superconducting cavity (Furuya *et al.*, 1996 ▸), the growth rates were estimated to be 12, 23 and 13 s^−1^, respectively.

The vacuum system is also crucial. In 5.0 GeV operation, it must satisfy the demands of absorbing the thermal load from synchrotron radiation and providing adequate radiation shielding. On the other hand, in 2.5 GeV operation, the effects of impedance and scattering due to residual gas become more pronounced, necessitating a system with both low impedance and high vacuum quality. Therefore, beam duct materials must exhibit high radiation-shielding performance, high electrical conductivity and low outgassing. Considering maintainability, oxygen-free copper is a promising candidate. Furthermore, given the relatively small bore diameter of the beam duct, the adoption of an ante-chamber design (Suetsugu *et al.*, 2005 ▸; Ganter *et al.*, 2024 ▸) to reduce the synchrotron radiation power density and the application of non-evaporable getter (NEG) coating (Benvenuti *et al.*, 1998 ▸; Jin *et al.*, 2023 ▸) to achieve low photon-stimulated desorption rates and high pumping speeds are also essential.

### 3.2. Insertion device

The parameters and brilliance spectra of typical insertion devices are listed in Table 2 ▸ and shown in Fig. 3 ▸, respectively. For single-energy operation, the parameters of the insertion devices are optimized to achieve the highest brilliance under the required wavelength and polarization conditions. In the case of energy-switching operation, additional care must be taken to ensure that the radiation power during high-energy operation is not excessive. Furthermore, during low-energy operation, the ratio of the radiation loss from the insertion devices to that from the bending magnets becomes significant (see Section 2.2). Therefore, changes in electron beam parameters due to changes in the insertion device gaps should be examined. For instance, assuming the installation of a total of 50 insertion devices (8 units each of the 4 m IVU, 1 m IVU, EPU APPLE-II type and EPU six-row type; 16 units of OV MPW; and 2 units of SC MPW) with undulator gaps set randomly and wigglers fixed at minimum gap, the change in emittance was estimated to be 8 pm rad at 2.5 GeV and 12 pm rad at 5.0 GeV (standard deviations, equivalent to 4% and 1% of the natural emittance), respectively. If this becomes an issue, the implementation of compensatory wigglers will be considered.

### 3.3. Beamline

**Figure 5:**
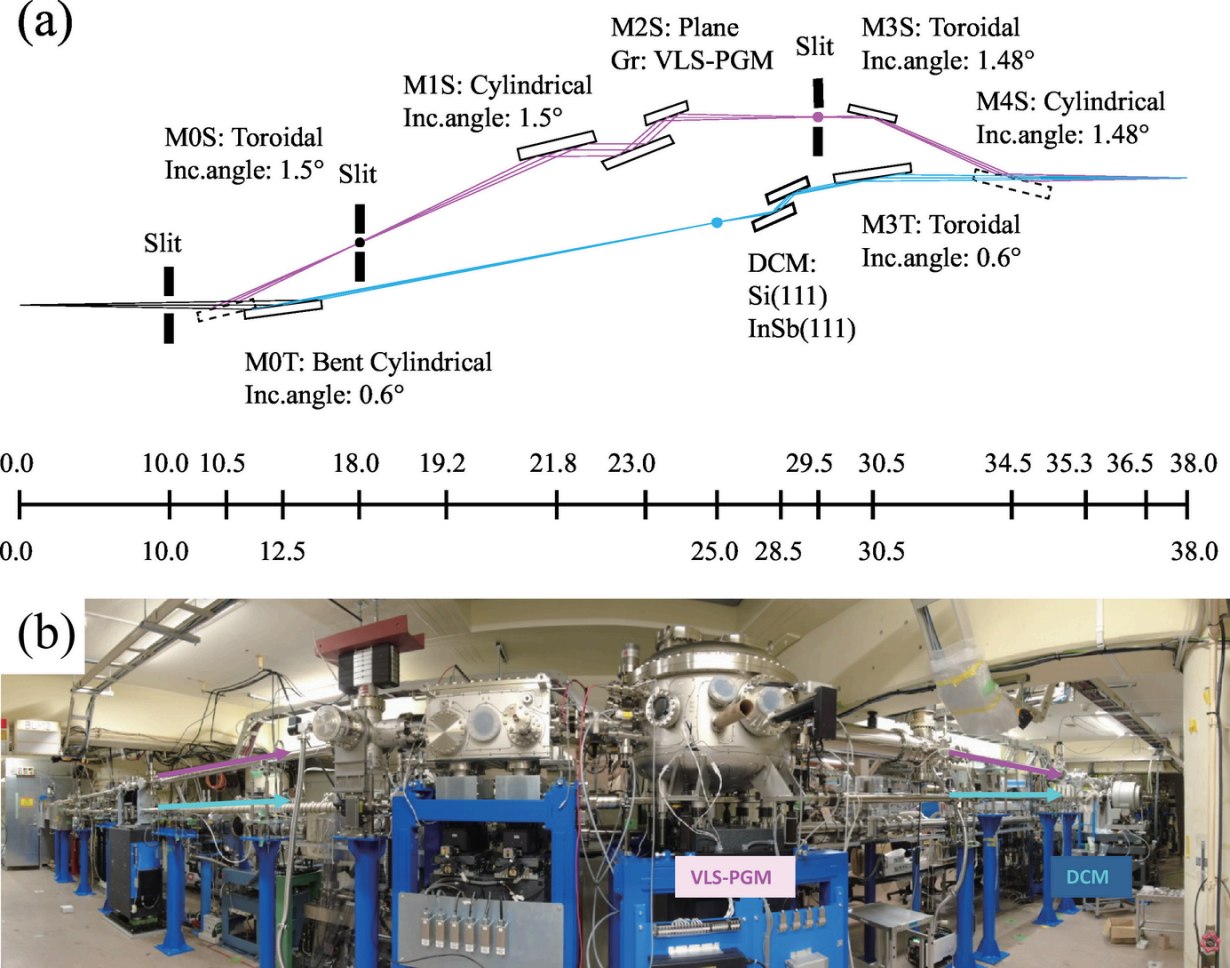
(*a*) Layout and (*b*) photograph of the wide-wavelength-range soft X-ray beamline at PF BL-12A (side view). Magenta and cyan lines represent the soft and tender X-ray paths, respectively.

Switching between 2.5 GeV and 5.0 GeV significantly expands the wavelength range accessible with a single undulator. As shown in Fig. 3 ▸(*a*), by placing two undulators for different wavelength ranges in a long straight section, introducing high-brilliance light from 10 eV to 100 keV, spanning four orders of magnitude, into a single beamline becomes possible. Radiation shielding will be designed to meet the requirements for 5.0 GeV operation. Even when switching between 2.5 GeV and 5.0 GeV, no special modifications to beamlines are required if the wavelength range common to both energy modes is utilized. However, switching between multiple optical elements is essential when utilizing a wide wavelength range on a single beamline. The most critical component is the monochromator. When spanning the vacuum ultraviolet to soft and hard X-ray regions, switching between a grating monochromator and a double-crystal monochromator is necessary. In addition, depending on the requirements, multiple diffraction gratings for the grating monochromator or multiple crystals for the double-crystal monochromator need to be switched. A higher-order harmonics suppression mechanism, such as mirrors coated with multiple elemental stripes, is also required to accommodate a wide wavelength range. For measurements, the detector will be switched as needed to one suitable for the wavelength in use. The newly constructed BL-12A at PF, the wide-wavelength-range soft X-ray beamline, is designed to irradiate the same sample position with a wide wavelength range from 50 eV to 5 keV by selecting either the grating monochromator path or the double-crystal monochromator path through switching of the first and final mirrors (Fig. 5 ▸). Demonstration experiments for the related beamline technologies are planned.

### 3.4. Switching time

Fluctuations in the beam position associated with switching between 2.5 GeV and 5.0 GeV could pose challenges for measurements. During the switching process, changes in the thermal load on components, such as electromagnets and beam ducts, as well as the expansion and contraction of the building, are expected to induce gradual variations in the electron beam orbit. Based on experience with existing facilities, it takes at least one day to sufficiently stabilize the magnetic fields and approximately three days for the entire accelerator to reach a stable state. These fluctuations may also affect the stability of mirrors and monochromators in the beamline. One approach to mitigate thermal fluctuations is to increase the capacity of air conditioning and cooling systems; however, this would significantly increase power consumption. To minimize the switching time, a high-speed orbit stabilization system consisting of a minimum of 14 electron beam monitors per cell, fast corrector magnets and a fast digital signal processing system are required (Safranek, 1997 ▸; Harada *et al.*, 2009 ▸). Furthermore, at least two synchrotron radiation beam monitors are installed on each beamline. Using the information (position and angle) obtained from these monitors, coordinated local feedback is implemented for both the storage ring and the beamlines. Appropriate feedback enables rapid switching. Considering thermal stability, the target switching time is within a few hours. An operating mode for repeated switching within a few to ten minutes will also be explored.

## 4. Science cases for the ESSR

A key feature of the ESSR is its ability to utilize a wide wavelength range on a single beamline, which, even considering the time required for energy switching, provides significant advantages for the measurement of samples under specialized conditions. For example, when a large apparatus is required to generate experimental conditions or perform measurements (*e.g.* superconducting magnets, high-power lasers, long beamlines) or when stringent safety managements are necessary (*e.g.* radioactive elements, pathogens, high-pressure gases), there is no need to install these large-scale systems for multiple beamlines. Additionally, in experiments where samples are synthesized and measured *in situ* or where external fields are applied to samples during measurements, it is often quite difficult to fully replicate the synthesis conditions or external field conditions. More accurate insights can be obtained by enabling multiple types of measurements for the sample in the same state on the same beamline. Traditional synchrotron experiments have often been conducted under the assumption that sample conditions are reproducible. However, it will become increasingly important to achieve a more precise understanding of the sample state, including subtle differences in synthesis conditions and external field conditions. Below are examples of science cases in which reproducing exact sample states is particularly challenging.

### 4.1. Research on magnetic thin films through element-selective analysis of structure and magnetic states

Magnetic thin films exhibit unique magnetic properties such as perpendicular magnetic anisotropy. Furthermore, by appropriately layering different types of thin films, including non-magnetic materials, they demonstrate intriguing phenomena such as the giant magnetoresistance effect and exchange bias effect, which are applied in the field of spintronics technology (Freeman & Wu, 1991 ▸; Jussila *et al.*, 2023 ▸). One of the origins of these phenomena is the atomic arrangements (structures) unique to nanoscale thin films, which differ from those of bulk materials. Although the structure and magnetism of thin films have been extensively studied using various techniques, the magnetic properties of thin films can change significantly in response to minor differences in the structure or chemical state. Therefore, it is critically important to reliably observe both the magnetic state and structure of the same sample. However, complete reproduction of synthesis conditions is quite difficult, and attention must also be paid to potential changes in the sample after synthesis. In this context, the ability to measure both the magnetic state and structure on a single beamline is highly advantageous in ensuring the identity of the sample.

The analysis of magnetic states requires observing the orbitals responsible for magnetism using X-ray magnetic circular dichroism (XMCD) and X-ray magnetic linear dichroism (XMLD) (Stöhr, 1999 ▸; Miyawaki *et al.*, 2009 ▸; Sakamaki *et al.*, 2016 ▸). In the range of several hundred eV to 4 keV, there are absorption edges for a wide variety of elements that constitute magnetic thin films, including the *L* absorption edges of 3*d* and 4*d* transition metals and the *M* absorption edges of rare-earth elements and 5*d* transition metals. By switching between 2.5 GeV and 5.0 GeV, XMCD, which requires circularly polarized light, can be measured for these elements using a single APPLE-II type undulator source [Fig. 3 ▸(*b*)]. For the structural analysis, extended X-ray absorption fine structure (EXAFS) utilizing linearly polarized light can be employed to determine the bond lengths along both the vertical and in-plane directions of the film. This enables the acquisition of structural information directly linked to the emergence of unique magnetic properties, such as lattice matching/mismatching at the interfaces between different types of thin films and the resulting structural distortions (Lee *et al.*, 1981 ▸; Miyawaki *et al.*, 2009 ▸; Sakamaki *et al.*, 2016 ▸). The *K* absorption edge of 3*d* transition metals and the *L* absorption edge of 5*d* transition metals, which fall within the 2–12 keV range, can also be accessed using the same undulator source [Fig. 3 ▸(*b*)]. For 4*d* transition metals, the *L* absorption edges are located between approximately 2 and 4 keV. However, the *L*_3_ and *L*_2_ absorption edges are close to each other, making EXAFS analysis more challenging. Installing an additional in-vacuum undulator [Fig. 3 ▸(*a*)] on the same beamline to enable the use of the *K* absorption edges (approximately 18–27 keV) would be highly advantageous.

### 4.2. Research on the structure of liquids and glasses through wide-wavenumber range scattering and electronic state observation

Structural disorder in liquids and glasses is fundamental to their uniformity, whereas structural order such as short-range and network structures is key to their mechanical, physical and chemical properties. The detailed structure of liquids and glasses has attracted interest from both scientific perspectives, focusing on the interplay of disorder and order, and industrial perspectives, aiming to enhance the performance of conventional glasses and develop novel glass materials. However, reproducing the same sample state is quite difficult, as liquids tend to experience compositional instability due to evaporation, and glasses undergo structural relaxation over long timescales. Consequently, conducting measurements on the same sample as quickly as possible is crucial.

The most common technique for measuring liquid and glass structures is total scattering up to high-*Q* regions (> ∼25 Å^−1^) using high-energy X-rays (> ∼50 keV) (Egami & Billinge, 2012 ▸). Recently, structural analysis across a broad scale has been attempted by combining total scattering data with small-angle scattering data obtained by using ∼10 keV X-rays (Sato *et al.*, 2018 ▸; Benmore *et al.*, 2020 ▸). Since liquids and glasses can incorporate various elements, combining measurements such as XAFS or anomalous (resonant) scattering, which selectively provide the electronic state and structural information of specific elements, is expected to yield more detailed structural insights. Using an in-vacuum undulator makes it possible to access the 4–100 keV range at 5.0 GeV as well as the 1–4 keV range at 2.5 GeV [Fig. 3 ▸(*a*)]. This range includes the *K* absorption edges of elements such as Na, Mg, Al, Si, P, S, Cl, Ar, K and Ca, which are abundant in the Earth’s crust and are important in geoscience and materials science. Even for these common elements the valence states and coordination numbers within liquids and glasses are not fully understood. By performing XAFS, anomalous scattering or chemical imaging using ptychography on the same beamline as the total and small-angle scattering measurements, it becomes possible to elucidate not only the structures but also the element-selective local electronic states of liquids and glasses.

## 5. Selection of circumference and energy for a versatile synchrotron light source suitable for long-term operation

In designing a synchrotron facility, determining the electron energy and ring circumference, both of which directly impact the performance and cost, requires careful consideration. In this study, we proposed a 750 m circumference storage ring designed to be switchable between operating energies of 2.5 GeV and 5.0 GeV. This ring configuration includes a diverse set of straight sections—15 sections of 10 m, 15 sections of 5 m and 30 sections of 2 m (Table 1 ▸ and Fig. 2 ▸)—designed to accommodate a wide variety of applications. If the circumference were reduced by half to 375 m, which is suited for 2.5 GeV, the number of straight sections would be roughly halved, and the emittance would increase approximately eightfold [Fig. 4 ▸(*a*), inversely proportional to the cube of the circumference]. In this case, the radiation loss from the bending magnets, which increases inversely with circumference, also increases significantly (see Section 2.2). Reducing the circumference entails additional considerations: Certain straight sections are necessary for the injection system and RF cavities regardless of the circumference, meaning that, although the construction cost would decrease substantially, it would be more than half of the original cost. In addition, the number of installable beamlines would be less than half. Therefore, when the circumference is small, the advantages of superconducting cavities become more significant because of their high cavity voltage in addition to low beam-coupling impedance (see Section 3.1). With respect to power consumption, a halved circumference would result in reduced power consumption for 2.5 GeV/500 mA operation but increased consumption for 5.0 GeV/200 mA [Fig. 4 ▸(*b*)]. The proposed design allows flexibility in the distribution of the operating time between 2.5 GeV and 5.0 GeV, enabling adaptation to shifts in the demand for specific wavelength ranges. Thus, the parameters being considered are advantageous for long-term operation and excel in the versatility essential for advancing synchrotron science.

## 6. Related literature

The following references, not cited in the main body of the paper, have been cited in the supporting information: Braun *et al.* (2021 ▸); Reichert (2017 ▸); Sekiguchi *et al.* (2013 ▸).

## Supplementary Material

Supporting Table 1. DOI: 10.1107/S1600577525005363/yn5125sup1.pdf

## Figures and Tables

**Table 1:** Tentative parameters for the ESSR

Energy (GeV)	2.5	5.0
Circumference (m)	749.5	–
Lattice	Double DDBA	–
Normal cell number	15	–
RF voltage (MV)	1.6	6.5
Bucket height (%)	4.6	5.0
Energy loss (MeV/turn)	0.151	2.412
Momentum compaction	1.306 × 10^−4^	–
Betatron tune, ν_*x*_/ν_*y*_	47.865/16.655	–
Damping time, *x*/*y*/*z* (ms)	58.2/82.9/52.6	7.28/10.36/6.58
Storage current (mA)	500	200
Natural emittance (nm rad)	0.212	0.846
Energy spread	5.04 × 10^−4^	1.01 × 10^−3^
Natural bunch length (mm)	1.929	2.803

**Table:** 

ID type	Polarization	Periodic length (mm)	*K*-value (magnetic field)	Minimum gap (mm)	Total length (m)	Wavelength range (keV)	Orbital amplitude (µm) @2.5/5.0 GeV
IVU	H	20	2.1	4	4, 1	1–100	–
EPU (APPLE-II type)	H/C/V	48	4.0	12	4	0.2–15	–
EPU (six-row type)	H/C/V	160	7.6	12	4	0.01–5	–
OV MPW	H	120	(>1 T)	12	∼0.5	White	∼50/∼25
SC MPW	V	80	(∼3 T)	40	∼0.5	White	∼60/∼30

**Table:** 

	Maximum energy loss (keV/turn)		Emittance change (pm rad)	
ID type	@2.5 GeV	@5.0 GeV	@2.5 GeV	@5.0 GeV
IVU	20.0, 5.0	80.1, 20.0	−18.0, −4.8 (0.2)	−19.3, −4.9 (−2.4)
EPU (APPLE-II type)	12.9	51.5	−11.9/−6.6/−6.9	−12.5/−6.8/−7.1
EPU (six-row type)	5.1	20.5	−4.9/−3.7/−1.0	−5.0/−3.8/−1.0
OV MPW	1.9	7.6	−1.8 (0.1)	−1.9 (−1.0)
SC MPW	22.8	91.2	−20.3 (36.1)	−21.9 (8.5)

Values in parentheses are for the short straight section.
