# Supplementary material for: Conceptual design of the energy-switchable storage ring as a high-brilliance light source over a wide wavelength range
Source: J Synchrotron Radiat. 2025 Jul 21;32(Pt 5):1143–51. doi: 10.1107/S1600577525005363 (PMC12416436; doi:10.1107/S1600577525005363)
Supplement: Supplementary file 1 [file s-32-01143-sup1.pdf]

JOURNAL OF SYNCHROTRON RADIATION

Volume 32 (2025)

Supporting information for article:

Conceptual design of the energy-switchable storage ring as a high-brilliance light source over a wide wavelength range

Tomoko Sato, Nobumasa Funamori, Kenta Amemiya, Noriko Usami, Takuji Ohigashi, Ryoma Kataoka, Nobutaka Shimizu, Hirokazu Tanaka, Hironori Nakao, Yusuke Yamada, Daisuke Wakabayashi, Takashi Obina, Masahiro Adachi, Yukinori Kobayashi, Yoshito Shimosaki, Yasunori Tanimoto, Kimichika Tsuchiya, Kentaro Harada, Naoto Yamamoto and Noriyuki Igarashi

Table S1 Power consumption

	**PF**	**PF-AR**		**SLS[1]**	**ESRF[2]**	**SPring-8[3]**
E [GeV]	2.5	5.0	6.5	2.4	6.0	8.0
C [m]	187	377		288	844	1436
I [mA]	450	50	50	400	200	100
$P_{facility}$ [MW]	3.3	4.4	6.5	3.3	10.0	18.3
magnet	1.8	2.3	3.3	0.9	1.9	3.9
RF	0.5	1.1	2.0	1.3	2.8	5.6
others_run	0.3	0.3	0.5	1.1*	2.6	2.9
others_base	0.7	0.7	0.7	-	2.7	5.9

*This value includes the others_base power consumption.

[1]Braun, H. *et al*. (2021). SLS 2.0 storage ring. Technical design report. Villigen PSI, Switzerland: Paul Scherrer Institut, 293 p. Report No.: 21-02.

[2]Reichert, H. (2017). *4th Workshop Energy for Sustainable Science at Research Infrastructures*, ELI-NP, Magurele, Romania.

[3]Sekiguchi Y., Tobinaga T., & Uenishi M. (2013). *Proceedings of the 10th Annual Meeting of Particle Accelerator Society of Japan*, pp.1239-1242. Nagoya, Japan.
